# Towards Emotion Detection in Educational Scenarios from Facial Expressions and Body Movements through Multimodal Approaches

**DOI:** 10.1155/2014/484873

**Published:** 2014-04-22

**Authors:** Mar Saneiro, Olga C. Santos, Sergio Salmeron-Majadas, Jesus G. Boticario

**Affiliations:** aDeNu Research Group, Artificial Intelligence Department, UNED, Calle Juan del Rosal 16, 28040 Madrid, Spain

## Abstract

We report current findings when considering video recordings of facial expressions and body movements to provide affective personalized support in an educational context from an enriched multimodal emotion detection approach. In particular, we describe an annotation methodology to tag facial expression and body movements that conform to changes in the affective states of learners while dealing with cognitive tasks in a learning process. The ultimate goal is to combine these annotations with additional affective information collected during experimental learning sessions from different sources such as qualitative, self-reported, physiological, and behavioral information. These data altogether are to train data mining algorithms that serve to automatically identify changes in the learners' affective states when dealing with cognitive tasks which help to provide emotional personalized support.

## 1. Introduction

Adaptive systems can be used to intelligently manage the affective dimension of the learner in order to foster the interplay that exists between the cognitive aspects of learning and affect [1]. In our research, currently framed within the MAMIPEC project [2], we aim at integrating cognition with user's emotions to provide adaptive learning, where a multimodal approach based on mining several input data sources (e.g., physiological data, keyboard and mouse interactions, explicit subjective affective information provided by learners, facial expressions recordings, etc.) is used for emotions detection [3] and modeling semantically affective recommendations that are used to deliver emotional personalized support [4].

To progress our research we have carried out a large-scale two-week experiment in our laboratory, which aimed at obtaining a database of emotional data in an educational context, from where to analyze the viability of inferring learning emotions. This was our first step to building a user model that considers student's emotions, to be used to better personalize the learner experience. In this paper, we focus on the emotion detection approach followed to extract affective information from the facial expressions and body movements recorded in the aforementioned observational study with the objective of enhancing the multimodal detection approach. In particular, we focus on reporting the methodology derived from a psychoeducational expert involvement in dealing with the problem of annotating with meaningful predefined tags changes in the affective states of the participants when visualizing recorded videos on their performance while dealing with cognitive tasks in a learning context. This methodology conforms to the data gathered from the learner global interaction, including their task performance and self-reported emotional reports. The ultimate goal is to use these annotations to train a data mining based system that can automatically identify user's affective state changes and, from them, provide the required affective support.

The paper is structured as follows. First, related works are introduced. Next, the experiment carried out is described. After that, the data gathered in a large-scale experiment are reported, which in turns supports the description of the methodology followed for the body movement and facial expressions detection. Following, we discuss the main challenges and difficulties found. The paper ends with some conclusions and the outline of future work.

## 2. Related Works

Humans display their emotions through different channels. Facial expressions, body movements, and physiological reactions are considered as elements of the nonverbal communication forms [5]. These information sources along with eye movements, tone of voice, posture, and spatial distance between two or more people, among others, are considered in the research field of “affective computing,” which allows computers to detect and recognize affect [6]. Tracking the user's affective state is required by the computing system to express and recognize affects and ultimately may be used to offer an adapted learning flow according to user's needs and context's features [7, 8]. In addition, the application of emotion recognition by analyzing nonverbal communication like body movements and facial expressions has a great potential for evaluating learners' states [9], because the emotion is detected and analyzed unobtrusively and automatically in real time using a video camera, thereby facilitating an engaging and optimal learning experience.

Nowadays the study of the analysis of universal facial expressions is a hot research issue. Different approaches can be used by observers to measure facial expressions [10]: (i) judgment-based approaches centered in messages communicated by facial expressions (e.g., a smile is categorized as happy) that can be analyzed by the observer; and (ii) sign-based approaches where the observer describes behavior by counting types and frequency of movements, such as how many times the face moves, and how long a movement lasts (e.g., a smile is categorized as an oblique movement of the lip corners, with a specific duration). Observers with a sign-based approach are supposed to function like machines and typically are referred to as “coders.” Some of the most relevant studies related to this later approach are those performed by Ekman and Friesen [11]. Their theory is based on the existence of six main universal human facial expressions: happiness, sadness, anger, fear, surprise, and disgust. These authors developed the Facial Action Coding System (FACS) to categorize facial expressions [11]. Through this system the movements on the face are described by a set of action units (AUs) with a muscular basis. These researchers analyzed facial expressions by detecting facial gestures and measuring the amount of facial movements. Other similar coding systems are EMFACS [12], MAX [13], AFFEX [14], and CANDIDE-3 [15]. Ekman et al. [16] presented also a database called FACS Affect Interpretation Dictionary (FACSAID), which allows translating emotion related FACS scores into affective meanings. However, according to other works [17], some of the facial action units included in these systems may not appear in meaningful facial expressions, as they describe any visually distinguishable facial action and do not concentrate on emotional expressions. Moreover, they are appearance-based and do not consider any information about cognitive process (which are very relevant in an educational scenario) associated with expressions.

More recently, facial expression analysis research has experienced important changes due to multiple factors such as the achievements in face detection, tracking and recognition, and the application of advanced computational techniques. In particular, many methods have been used for face tracking, which include measurement of optical flow [18], face identification, pose recovery and facial expression [19], models of image motion to recover nonrigid facial motion [20], active contours [21], robust appearance filter [22], probabilistic tracking [23], active [24] and adaptive [25] appearance model, image-based motion extraction [26], and multiple eigenspaces [27]. As a consequence of this extensive research work, different algorithms have been applied to facial expression recognition, such as Hidden Markov Models [28], Support Vector Machines [29], Neural Networks [30], Bayesian Network Classifiers [31], using other algorithms as Principal Component Analysis [32], and Linear Discriminant Analysis [33] to reduce the dimensionality of the data.

Furthermore, affective computing has undergone relevant changes in the emotions detection and recognition process regarding body movement and posture. In the last decade many researchers have revealed that body movement, posture, and gesture can be considered as indicators of affective states [34] playing a relevant role in the human emotional states interpretation [35]. There are several methods aimed to detect emotion showed by actors through body posture by using photos without 3D information. These photos are decoded by using low-level visual data [36]. Other researchers have built a complicated body posture representation system by capturing the position of different body parts generating a set of features (i.e., distance and angles between shoulder and head, etc.) [37]. However, these methods do not include an automatic process for generating the features needed to recognize emotions through body posture. None of the most used gesture emotion detection systems—such as the VICON system, which takes inputs from 3D positions of magnetic markers attached around joints and body parts [38]—apply automatic recognition techniques. There are different methodologies for coding body movement such as the Bernese system [39] and the most recent Body Action and Posture Coding System [40]. Often the researches categorize emotion expressions by making inferences from body movements but they cannot be replicated by others. In other cases, measures of movement at muscular level are used but the devices used limit the participant freedom [41]. Furthermore, video based automatic tracking techniques, which are considered less obtrusive methods, have a lack of capability to detect fine body movements [42]. Finally there are a great number of methods for coding body movement; but up to now, there is not a theoretical framework establishing which are the basic units of body movement to be used in emotion detection through body movement, posture, and gesture [43].

From the above related works follows that emotion detection through body movements is reduced because there are not available standard-based coding schemes for body movement, posture, and gesture expression equivalent to the FACS [40]. Another relevant issue to be considered for further studies is the improvement of the ecological validity. Ecological validity or external validity refers to whether or not one can generalize from observed behavior in the laboratory to natural behavior in the world [44]. Many studies to date have used static images of facial expressions and body movements, which do not represent the natural expressions for the corresponding emotional state showed in a real interaction context. To ensure ecological validity, it is essential to study the occurrence and nature of affect displays in situ, as they occur [45]. Although there exist databases of recordings dedicated to human emotional experiences [46, 47], these are mostly based on posed expressions and postures showed by actors or recorded in scripted situations that may not be entirely relevant in a learning scenario. Nevertheless, human emotion recognition involves not only facial and body motion but also other variables such as the cognitive process (memory, attention, etc.) developed by the subject and contextual information provided by the environment, among others [45].

According to this, a complete system for emotion recognition through facial and body movement analysis facilitating affective support during the real learning process is still an open research issue. This paper's objective is to report the methodology derived from a psychoeducational expert involvement in dealing with the problem of visualizing and annotating—with meaningful predefined tags—changes in the affective states of learners who were video-recorded during experimental learning sessions. The goal is to combine these annotations with additional affective information sources such as qualitative, self-reported, physiological, and behavioral information. These data altogether will be considered to train data mining algorithms that can automatically identify changes in the learners' affective states when dealing with cognitive tasks. In this way, appropriate affective personalized support from a multimodal detection approach can be provided. This system should dynamically analyze the reactions showed by the users in real learning and social context and be able to interpret these responses in terms of emotions, which are to be confirmed with the emotions reported by them while or just after performing the cognitive tasks.

## 3. Experiment Description

To detect emotions from users' interactions in an e-learning environment, we have designed a series of large-scale activities aimed to record multiple measures (qualitative, self-reported, physiological, and behavioral). Each of the activities was addressed to a different target audience (general public, high school kids, high school, and college youth) and involved different learning styles and difficulties while facing math problems. Three out of four were designed to be carried out individually [3]. The other one followed a collaborative approach [48]. In this paper we focus our analysis on individual experiments, thereof not considering additional variables (e.g., interdependence of other people, level of social competence, anxiety related with being better than others, etc.), which should be considered when emotion expressions are being analyzed in collaborative tasks. Moreover, collaborative scenarios provide information about cognitive process and strategies specifically involved in social contexts, which deserve a different analysis [49]. Furthermore, it can be mentioned that participants with disability were also involved in the experience [50] but this analysis also deserves future work since there are differences in the variables to be considered when people have disabilities (e.g., there might be morphological or functional alterations that imply that their facial expressions and/or body movements vary in terms of intensity, location, or duration; users of screen readers use the keyboard for browsing interactions, not just typing). All these experiments took place in the so-called Madrid Science Week (https://adenu.ia.uned.es/web/es/Proyectos/SemanaCiencia (in Spanish)). The experiments' goal was to check if the emotions elicited during the execution of the designed cognitive tasks can be detected with the technological infrastructure prepared and codified accordingly considering as input the combination of diverse sources for gathering emotional data from participants, including facial and body movement recordings with webcam and Kinect devices.

### 3.1. Description of Experimental Tasks

During the three individual activities (identified as act2, act3, and act4—act1 being the collaborative experiment), we proposed several tasks for the participants to be done individually. The structure of them was similar; the only difference was on the contents of the problems to better fit the targeted audience (i.e., general public and students of diverse levels). Additionally, some previous pilot studies were performed in order to verify the proper setting of the experiment for the activities, which included the technological infrastructure required. Sessions were scheduled every two hours, with a capacity for up to 4 participants at a time. In each experimental stand there was one researcher in charge of recording measures and supporting the participant along the session. An additional researcher was taking notes in an unobtrusively way about relevant physical movements of the participants. There was also a researcher guiding the activity in a synchronous way so all participants were jointly informed about the tasks to be carried out. However, no interaction within participants was allowed during the activity as this was designed in an individual manner. The activity was prepared in the accessible dotLRN e-learning platform [51]. Along with the physiological and behavioral recordings, participants filled out some personality trait questionnaires (i.e., Big Five Inventory—BFI [52], General Self-Efficacy Scale—GSE [53], Positive and Negative Affect Schedule—PANAS [54]) as these characteristics relate to how students respond to attempts to provide them affective scaffolding [55]. The data gathered from the interactions in the learning activity were complemented with emotional feedback collected directly from learners in terms of the Self-Assessment Manikin (SAM) scale [56] to measure emotions in a dimensional space [57], and emotional reports filled by the participant when the tasks were finished so they could report about their emotions, difficulties encountered, and strategies used to solve them.

### 3.2. Experiment Phases

Each activity was divided into several phases as described below in the same order as participants carried them out.

#### 3.2.1. Prestudy

In the prestudy, relevant information about the participants was gathered. Moreover, the required calibration was carried out. Three phases were defined here.

Phase 1 consisted in the welcome introduction to the participant, explaining in a very general manner (not to influence the experiment) the activity to be carried out. After that, the information consent form was given to the participants to be carefully read and signed if they agree on it. Here they were informed about the anonymous treatment of their information as well as their freedom to leave the experiment at any time. This information consent was approved by the ethical board of our university. All participants agreed to sign and none of them left the experiment. After the consent was signed, sensors were attached to the participant. These sensors consisted of disposable Ag/AgCl electrodes to get electrocardiogram signal, Pneumograph belt strapped around the chest, two 8 mm snap button style Ag/AgCl pellet in contact with index and ring fingers in the nondominant hand to measure galvanic skin response, and a temperature sensor attached to the nondominant wrist.

In Phase 2 demographic information was gathered, namely, gender, age, studies, occupation, illness related to heart and brain, physical activity, emotional control, and technological experience. Moreover, personality traits through the Big Five Inventory (BFI) and the General Self-Efficacy Scale (GSE) were also obtained.

Phase 3 was focused onthe emotions calibration. First, the physiological sensors baseline was recorded by asking the participant to relax for 2 minutes. Next, an expectative report (identified as T0) was filled in by the participants to collect their expectations for the activity. Here, besides analyzing the information written by the participants, emotional information can also be computed from their typing behavior as well as from a sentiment analysis of the text [3]. A polygraph-line task (identified as T1) was also used to calibrate the elicited emotional response in advance through a variety of questions that fluctuate from neutral to very lock-in ones. Finally, emotional images were selected from the International Affective Picture System (IAPS) [58] database—rated by a normative sample—covering the emotional dimensional spectrum to which participants were asked to rate valence (i.e., pleasure) and arousal (i.e., activation) with the 9 point SAM scale. This task was identified as T2.

#### 3.2.2. Study

Phase 4 consisted of some mathematical problems. Three groups of problems were given to the participants. The first group (identified as T3) contained simple mathematical problems to see how each participant reacted while doing mathematical tasks. The second group of problems (identified as T4) was of an equivalent difficulty level but participants were told that they were even easier than previous ones and the time available for their completion was limited to 3 minutes. Due to implementation issues, when the time limit was over, the participant was not aware of this factor till she moved to the next page (either after submitting a problem response or a SAM's score). This implies that the length in T4 was in average a bit larger than 3 minutes, but in any case, not sufficient to finish the task and participants could only finish the action on the current page. The goal here was to try to elicit a certain level of frustration and stress. The third group (identified as T5) consisted of simple graphical logical series to try to elicit a relief feeling from previous tasks and thus helping the participant finish the whole session with sensation of joy and happiness.

The structure was the same for each group of problems. First, a question and four possible answers, with only one correct answer, were provided. Participant had to select one alternative and submit the response. Then, they were provided with explained feedback on the correct answer. After that, they were asked to fill in the SAM scale, similarly as done with the images task (i.e., T2). After each group of problems, participants were asked to write down (typing) how they had felt when doing the problems, what they were thinking, what problems did they cope with, and how did they dealt with them. This self-reporting was asked to understand and confirm the meaning of the expressions and movement detected in relation to the emotion felt by the participant. This was the emotional report assigned to each study task (identified as ER-T_*i*_, where *i* = 3, 4, 5).

#### 3.2.3. Poststudy

The poststudy was used to collect participants' feedback after the experience. In Phase 5, the physiological sensors' baseline was measured again. Participants were asked as in the beginning to relax themselves for 2 minutes in order to check the recovery of their physiological variables after the study tasks. Sensors were withdrawn after that. Subjective feedback from participants was collected with the Positive and Negative Affect Schedule (PANAS) as well as from an ad hoc satisfaction questionnaire. After that, participants were informed about the goals and details of the experiment, telling them about the purpose of the tasks carried out, debriefed, and dismissed.

### 3.3. Technological Devices for the Data Gathering Process

Focused on getting as much facial and body movements information as possible from participants, two different devices were used in order to record all the gestures produced during the session. First, high definition webcams were used to capture the participant's face, located at a distance of about 75 cm from it. The video was recorded with the Logitech Webcam Software provided with the camera, generating a Windows Media Video file with a resolution of 1280 × 720 and 15 frames per second (fps). Second, in order to export some facial information, Microsoft Kinect for Windows was used. From version 1.5, the Kinect for Windows SDK provides a face-tracking engine that processes the image capture by the device and tracks human faces. Two different applications were used to get data from the Kinect. The first one was the Kinect Studio, an application included with the Kinect for Windows SDK that allows recording not only the video captured by the Kinect, but also the depth information associated to it (the Kinect device is equipped with an array of IR lights that allows it to get a matrix of depth points that can be associated to the video, generating a kind of 3D video). The files generated by the Kinect studio containing video (with a 640 × 480 resolution and 20 fps) and depth information are stored in an  .xed file with an average size of 1 gb/min. The second application used was a program developed by a partner in the MAMIPEC project (University of Valencia, Spain), which exports on the go (when a human face is detected) all the features extracted by the face tracking engine, including 100 characteristic points delimiting face, lips, nose, eyes and eyebrows outlines, and head pose (pitch, roll, and yaw), six Animation Units, and eleven Shape Units (which are a subset of the defined in the CANDIDE-3 model [15], the lines that recreate a 3D mask of the participant's face) and the timestamp of the values recorded. All these data are exported in a Comma Separated Values (.csv) file that contains several registries per second, having each one of these registries 1504 values.

Due to the high computational requirements to capture the video and data from Kinect, a stand-alone computer per stand was used. The computer where the webcam video was recorded was also used to obtain all the data from the physiological sensors (as aforementioned in Section 3). These sensors were connected to a J&J Engineering I-330-C2 device and controlled by the software provided with the device, that is, the J&J Engineering Physiolab. Another computer per stand was used to monitor the participant's desktop in real time with the realVNC software in order to know her progress during the learning tasks without disturbing her. The computer used by the participant (this makes 4 computers per stand) was also recording data. Specifically, a video with the participant's desktop (using CamStudio Portable) as well as the participants' interactions, running a hidden key-logger and mouse-tracker that records all the keystrokes, mouse clicks, and mouse movements during the session with their corresponding timestamps.

One computer in the lab was also configured as a Simple Network Time Protocol (SNTP) time server in order to synchronize the system time from all the computers used in the experiment to have all the timestamps synchronized, but the J&J Engineering Physiolab captured data, as the J&J, did not store the system time with the signals recorded. To solve this issue, timestamps were taken manually by each stand researcher to set, into the values, the beginning of the different tasks done in each session by each participant. The desktop recordings were useful to reproduce the participants' interaction with the computer, as commented in Section 4.1. In turn, collected keyboard and mouse interactions were processed in order to obtain indicators that correlate with emotional states [59].

75 participants (including 3 with accessibility requirements) took part in the three individual activities and 4.05 TB of data were collected in the following files for each participant: (i) a video of the participant's face recorded by the webcam, (ii) an  .xed file generated by the Kinect studio containing the video of the participant's face and the deep data of that video, (iii) a  .csv file containing all face information provided by the Kinect SDK with the timestamp of each registry generated, (iv) a  .csv file with all the keystrokes (key press and release) performed by the participant and another with all the interactions made with the mouse (button clicks and releases and mouse movements), both with the time of each interaction in it, (v) an  .xls file with all the physiological signals recorded and the timestamps added during the experiment, (vi) a video of the participants' desktop during the experience, (vii) several  .csv files with the participants' answers to the tasks in the learning platform, (viii) an  .xls file with the outcomes from the personality questionnaires and gathered demographic data, and lastly (ix) a spread sheet with the notes taken by the observer about relevant physical movements of participants.

## 4. Methodology for Facial Expressions and Body Movements Detection

As aforementioned, in this paper we focus on reporting the methodology proposed to detect facial expressions and body movements associated to emotions elicited during an individual learning activity, where cognitive processes (such as decision making and problem solving) are involved. A psychoeducational expert annotated collected data from the videos recorded in the experiment with meaningful predefined tags. She has more than 15 years of experience in learning disabilities personalized support, as well as in applying learning strategies (including emotions management) to improve the educational processes.

### 4.1. Annotation Process

**Figure 1:**
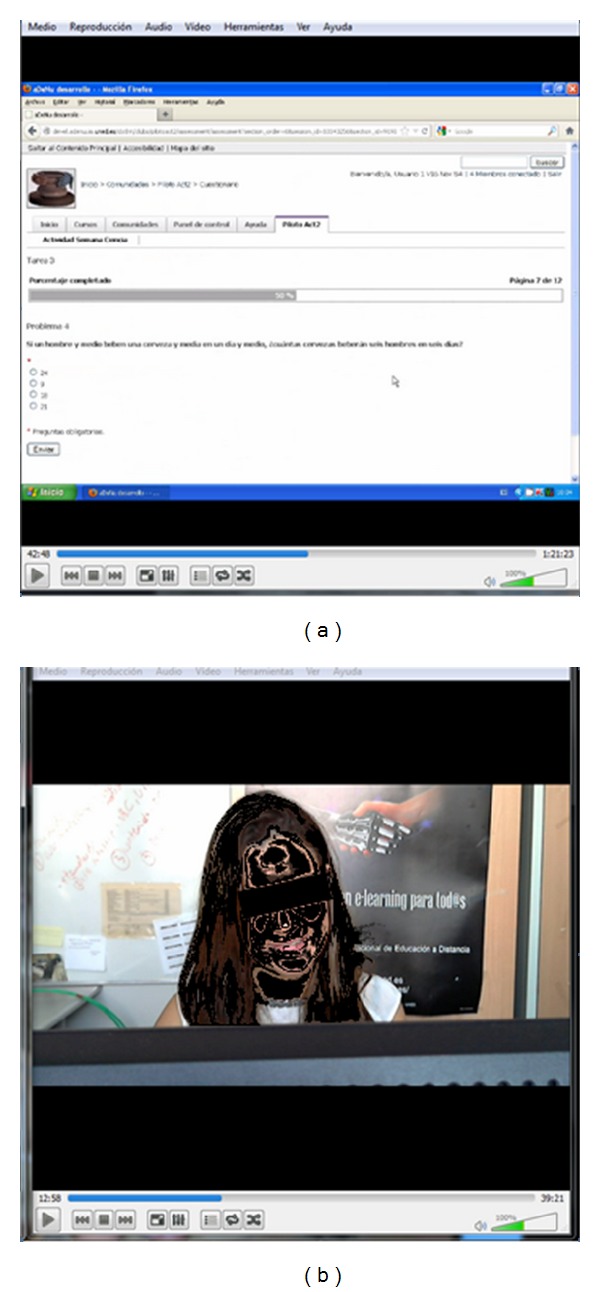
Desktop (a) and face (b) recorded videos for a participant simultaneously played on the experts computer to annotate the facial expressions detected when participant carried out a specific task. Note that face has been blurred to assure participant's anonymity as guaranteed in the signed information consent form.

A mixed (judgment and sign based) approach was followed to codify emotions associated with facial expressions and body movements. The expert gathered information about facial expressions and body movements associated to specific activities by viewing in a synchronized way two interfaces, (see Figure 1). Interface 1 (Figure 1(b)) shows the participants' facial expressions and body movements from the video (both image and sound). Interface 2 (Figure 1(a)) shows the current task performed by the participant in her computer's desktop.

The videos average length is between 50 and 70 minutes depending on the time spent by the participant to solve the proposed tasks. The duration and complexity of the video annotation process depends on several factors, such as the number of facial and body movements carried out by the learner and type of movement (an isolated movement or a movement involving different body parts such as the face, shoulder, etc.). Taking into account the complexity of the annotation process an extensive spreadsheet was designed and used for collecting the affective information from the two interfaces about the main features involved in the emotional expressions while the educational activity was performed. The corresponding timestamps were assigned to each task.

Another researcher (with less experience on facial emotions recognition) was asked to use this annotation methodology in a random sample of the videos to check that the coder's expertise did not bias the affective information obtained. After comparing the tags given by both experts, they tagged the same features. However, as expected, the expert was able to identify more relevant features than the nonexpert. After this verification, we felt reasonably confident about the tagging obtained.

Next, we comment on the relevant emotional features collected in the spreadsheet, which can be tagged from each of the two interfaces, as well as from the participants' global interaction.

#### 4.1.1. Data Gathered from Interface 1: Participants' Facial Expression and Body Movements

In order to categorize the facial expressions and body movements showed by the participant, the expert proposed and used the following fields.

- *Location:* a classification is made about specific facial actions and body movements. The actions are listed by general location (face, head, shoulders, hands, and trunk) or more specific locations (eyes, eyebrows, nose, lips, forehead, and neck) when appropriate.
- *Type of movement:* different types of movements were identified in face and body, such as turns, contractions, and deformations.
- *Movement direction:* coded options were in front, behind, right, left, up, and down. When several body parts are involved, a combined movement was identified instead of the direction.
- *Intensity of movement:* movements with a medium or high level of intensity were identified by the expert to highlight relevant moments where detailed attention should be put in the data mining analysis.
- *Repetitions of movement:* it is computed in terms of the number of movements carried out during the length of the task (obtained from Interface 2).

#### 4.1.2. Data Gathered from Interface 2: Learning Tasks

The information shown in the learning environment (i.e., participant' desktop) during the learning tasks was tagged by the expert as follows.

- *Task:* it is the specific task and mathematical problem that the participant was solving when the facial expressions or body movement was detected.
- *Result:* it is the result obtained by the participant in the task when a face and/or body movement was detected. This is qualitative information about the task result, which is to be enriched with the quantitative data collected in the learning platform for each of the tasks carried out. The result of the task is very relevant for the coding because it relates to the participant's cognitive process. Participants can show different body postures and facial expressions depending on the results obtained (wrong or right answer). Thus, this information is useful to categorize the associated emotion in an educational environment.
- *Task duration:* the starting and ending time of each task were written down. In this way, the number of movements detected can be properly compared when the lengths of the tasks are not the same.
- *Emotional reports:* the expert annotates not only the relevant information facilitated in the emotional reports by the participant about feelings, difficulties encountered, and the strategies used to solve them, but also the way she does the reporting, for instance, the participant writing speed, if she is thinking about the answer for a long time, and so forth.

#### 4.1.3. Data Gathered from Participants' Global Interaction

In addition to the labeling of the relevant events when visualizing Interfaces 1 and 2, the expert analyzed participants' global interaction throughout the learning activities (including the information facilitated in the emotional reports provided after each tasks) aimed to classify them into different categories related to the learning process. Given the lack of agreement in the community about a single vocabulary of emotion terms, the expert selected from the W3C Emotion Markup Language specification (EmotionML) [60] some of the most suitable categories according to the research activity described in this paper. A 5-point Likert scale was used to classify in a global way each participant in all the categories mentioned. The categories considered were the following.

- *Excited/Unexcited:* participants were categorized as excited when they showed an arousal state with a high rate of facial and body movements or they informed this state in the emotional report filled after the tasks.
- *Resolute/Confused:* participants were categorized as resolute when they did not hesitate to give the response and they did not modify the option chosen at any time, or they confirmed in the emotional report that they felt secure when delivering their responses.
- *Concentrated/Distracted:* participants were categorized as concentrated when they were focused on the task ignoring the possible distracting elements of the environment (i.e., other participants, researchers, noises, etc.).
- *Worried/Unworried:* participants were categorized as worried when after they finalized a task, they informed in the emotional report that they were worried by the obtained results.
- *Interested/Uninterested:* participants were categorized as interested when they tried to solve all the tasks, devoting time to looking and taking care of the feedback provided, avoiding random responses, and so forth. Participants were also included in this category when they informed in the emotional report about their interest in the task and their results showed the behaviors previously described.
- *Relaxed/Nervous:* participants were categorized as nervous when they showed a high rate of facial and body movements related with this emotional state (i.e., frowning, biting their lips, etc.). They were also included in this category when they confirmed this state in the emotional report.

### 4.2. Emotion Codification Process

Facial expressions and body movements detected by the expert in each participant as well as from the emotional reports and tasks carried out can be codified as discussed below.

#### 4.2.1. Facial Expressions

Information from facial expressions showed by the participant is codified using the Face Tracking SDK's face tracking engine. This tool analyzes input from a Kinect camera, deduces the head pose and facial expressions, and makes that information available to an application in real time. The output of the Face Tracking engine contains the following information about a tracked user.

**Figure 2:**
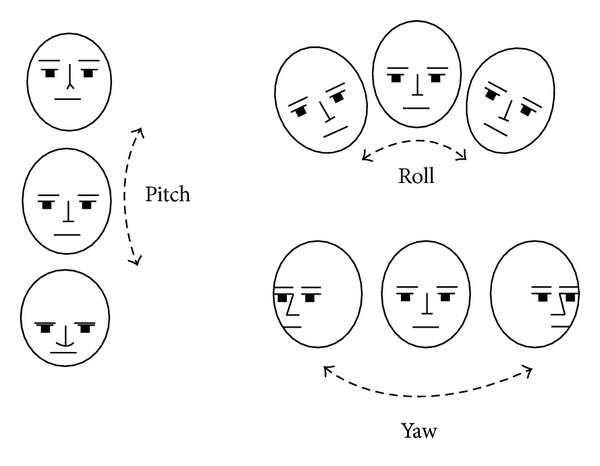
Head pose angles. The angles are expressed in degrees, with values ranging from −180 degrees to +180 degrees. This figure has been extracted from the Face Tracking SDK Kinect for Windows available at http://msdn.microsoft.com/en-us/library/jj130970.aspx.

**Figure 3:**
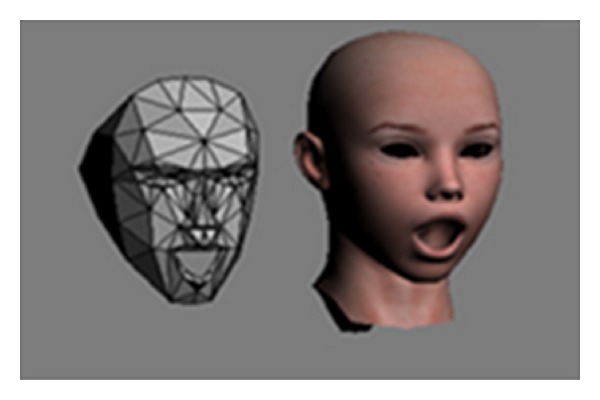
AU1—Jaw Lowerer Each AU is expressed as a numeric weight varying between −1 and +1. This figure has been extracted from the Face Tracking SDK Kinect for Windows available at http://msdn.microsoft.com/en-us/library/jj130970.aspx.

- *2D points:* The Face Tracking SDK tracks 87 2D points defined in the coordinate space of the RGB image (in 640 × 480 resolution) and returned from the Kinect sensor.
- *3D head pose:* The* X*,* Y*, and* Z* position of the user's head are reported based on a right-handed coordinate system. Translations are in meters. The user's head pose is captured by three angles: pitch, roll, and yaw (see Figure 2).
- *Animation and Shape Units:* The Face Tracking SDK results are also expressed in terms of weights of six Animation Units (AU) (see Figure 3) and 11 Shape Units (SU), which are a subset of what is defined in the CANDIDE-3 model. The SU estimate the particular shape of the user's head: the neutral position of their mouth, brows, eyes, and so on. The AU are deltas from the neutral shape that can be used to morph targets on animated avatar models so that the avatar acts as the tracked user does.

#### 4.2.2. Body Movements

Several methods are available for coding body movement in nonverbal behavior research, but as aforementioned in the related works there is no consensus on a reliable coding system that can be used for the study of emotion expression [61].

To progress on this issue, the psychoeducational expert proposed to determine the upper body movements in terms of posture and gesture, movement direction, and intensity and frequency. In addition, the expert added information if a specific gesture and posture are related with an emotional reaction showed by the participant in a specific moment.

#### 4.2.3. Emotional Reports

The expert analyzed if the information facilitated by the participant is in accordance with the facial expression and body movements showed during the task. This is valuable knowledge that can be used to

- confront the facial expression detected and categorized by the expert with the real emotion felt by the participant, and
- analyze the difficulties encountered by the participant and strategies applied to solve them in order to define the future affective support to be provided in each situation.

### 4.3. Analysis of Results from Annotation and Coding Processes

Up to now, the recordings of all the participants of the first stand for each of the three individual activities have been analyzed, which corresponds to 19 participants. A total of 15 hours, 15 minutes, and 55 seconds of video were watched by the expert for these 19 participants. For the tagging process, the expert had to stop the video at the relevant moments, identifying and coding the movement and writing it down in the predesigned spreadsheet. This has required in average an amount of time similar to three times the video recording (about 48 hours, 2 full days nonstop). The expert did not watch all the set of videos one after the other, but she did only one per day to keep attention focused. In this way, we can estimate that 1 minute of video recorded has required 3 minutes for the tagging process. To reduce this time, and based on the annotation process carried out, we have designed an annotation tool, as commented later in the future works section.

During the annotation process, the expert filled out the spreadsheet with the information described in Section 4.1. After that, the coding described in Section 4.2 was carried out. In this section, we report the findings currently identified regarding the study tasks (i.e., T3, T4, and T5) in Phase 4, which are the ones that properly relate to the cognitive processes carried out by the participants during the activity. For the analysis, we have combined the expert annotated data with the information gathered from the learning platform regarding the scoring for the tasks as well as the average SAM values for the valence and arousal dimensions given by the participants after answering each problem. In this respect we found a trend in the sense that participants with a large number of correct problems have higher values in the SAM's score for the valence, which suggests that they perceive the tasks as more pleasant when they answer correctly. This trend was not detected in arousal.

From the analysis of these data, several noteworthy issues were identified that refer the annotated movements' description to the task duration, task difficulty, and the codification process.

#### 4.3.1. Task Duration

To start the analysis, we comment on the amount of movements detected and the length of each study task.  Table 1 shows the amount of movements detected by the expert in each of the study tasks (row 1) as well as the total time recorded (in seconds) for each of these tasks (row 2). With this information, the ratio of movements per second for each of the tasks can be computed (row 3). Also, considering that there were 19 participants analyzed, the average number of movements and task duration can also be computed (rows 4 and 5). Regarding the average duration per participant in T4, it has to be recalled (as commented in Section 3.2.2) that the length of T4 was longer than 3 minutes (and thus, the average is larger than 180 seconds) due to implementation issues.

**Figure 4:**
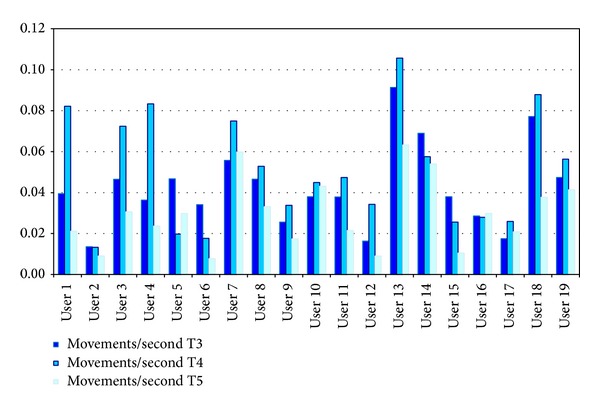
Movement rate showed by each participant (user_*i*_) along the three tasks analyzed (T3, T4, and T5).

More movements were detected in T3, but in T4 the ratio is larger. The duration of T4 is shorter because this task was limited in time to cause frustration and stress (see Section 3.1). In Figure 4 it can be seen that the rate of facial expressions and body movements (computed as the number of movements each participant carried out during the duration of the task) is larger in T4 (T4 is represented in the middle column in each group of bars if the figure) for almost all the participants analyzed. This suggests that to carry out a task with limited duration aimed to cause frustration and stress could be considered as a relevant issue impacting on the amount of facial expressions and body movement showed by the participants.

From Table 1, T4 and T5 had a similar average of body movements per participant (i.e., 9.47 and 9.68, resp.) even though T4 had a T4 had lower average lenght than T5. In T4 participants were told that they should carry out a set of mathematical problems with an equivalent difficulty level of exercise, even easier than previous ones, but the time available for their completion was limited and insufficient. In our view, this finding can suggest that* limited duration could elicit a certain level of frustration and stress in the participants and this emotional state produces a larger amount of facial and body movements*.

#### 4.3.2. Task Difficulty

Regarding the task difficulty level, some relevant issues can be also commented. Contrary to T3 and T4, T5 consisted of simple graphical logical series with a low difficulty level. The goal was to elicit a relief feeling from previous tasks and thus helping the participant finish the whole session with sensation of joy and happiness. In general, the amount of facial and body movements observed from the participants in T5 shows a lower rate (0.0283) than those showed in T3 (0.0387) and T4 (0.0487). This suggests that the task difficulty level is also a factor impacting on the facial and body movement rates. Our interpretation here is that* in tasks of medium or high level of difficulty such as T3 and T4, participants made more facial expressions and body movements while carrying out more complex cognitive processes involving, among others, attention and memory*.

**Figure 5:**
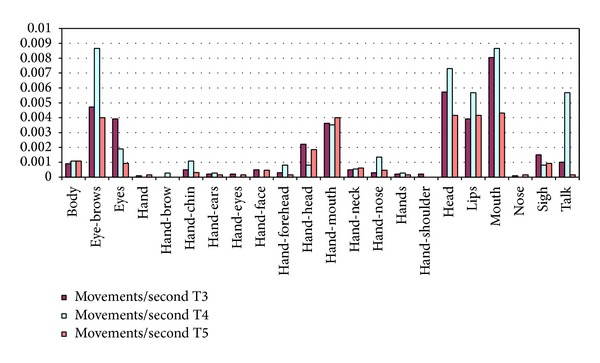
Mean of scores of specific facial and body movements showed by each participants along the three tasks analyzed.

We have also analyzed the types of face expressions and body movement and task difficulty level. During the annotation process, the expert noticed that there were some types of movement carried out by some participants more often than others. Furthermore the rate of these movements was higher when these participants were solving T3 and T4 considered with a medium or high difficulty level. By comparing the movements per second for each of the movement types in each of the tasks (see Figure 5), it can be seen that the movements of eyebrows is clearly larger in T4, the task where participants were stressed and frustrated due to the time limitation. It can also be seen that there is a large difference in the tag “talk.” This tag means that the participants made some aloud comment spontaneously. Most of these verbalizations in T4 were due to their surprise for the time being finished, as the task was limited. In this way, it seems that* certain face expressions such as eyebrows movements and verbalizations might occur more often when participants are stressed and frustrated as they are carrying out cognitive tasks with time limit.*

#### 4.3.3. Codification Process

According to the SDK codification system described in Section 4.2.1., the type of movement detected with a high frequency level was AU0—Upper L, AU1—Jaw Lowerer, AU2—Lip Stretcher, AU3—Brow Lowerer, AU4—Lip Corner Depressor, AU5—Outer Brow Raiser, 3D Pose—PITCH, ROLL, and YAW.

Some of these Action Units and 3D head poses such as those involving eyebrows (frown, raise), mouth (tighten, opening, biting, twisting sideways, and bowing down) and head (pitch, yaw, and roll) have been shown more often by some participants during T4, the task designed to generate stress and thus has a higher difficulty level and limited duration. From the first studies that try to relate facial expressions with specific emotional states [11], these expressions and poses seem related to concentrated, worried, and thinking states, among others. This supports the previous finding in the sense that it could be a relation between tasks with a high difficulty level where relevant cognitive process such as attention, memory, reasoning are functioning and specific types of movement mainly facial expressions showed by the learner while trying to solve the mathematical problems.

## 5. Discussion

The methodology proposed complies with several points that need to be addressed when measuring facial expressions and body movements as reported elsewhere [62]. In particular, following the proposal made by these authors, we remark the following issues that have been taken into account in the annotation methodology presented in this paper.

- A separate classification about specific facial actions and upper body movement has been developed, as shown by the items involved in the analysis of Interface 1.
- Only spontaneous facial actions and body movements have been considered (as they were recorded from participants doing learning tasks).
- Subjects participating in the research experiment belong to different population groups (gender, age, race, and functional diversity) which contributes to the generality of the approach proposed.
- Experts and beginners on facial emotion recognition have been included (to show that the emotion is easily detected and interpreted regardless of the coder's experience, culture, etc.).
- Facial expressions and body movements have been reported not only by analyzing the movement dynamically, but also determining their intensity and frequency.

In addition, some problems while gathering facial and body movements' information were detected.

- Regarding the software infrastructure, the psychoeducational expert analyzed the participant interaction on two different interfaces. Due to this fact, the data collection from body movements and facial expressions showed by each participant is a complex and time consuming process. Difficulties related to synchronization were encountered while she was detecting and extracting emotional information related with cognitive tasks performance. Synchronization of data gathered is a critical issue, addressed in the experimental design with the SNTP server and the manual labels, but still an open issue.
- Due to the sensitivity of the Kinect device, there are some factors that should be taken into account to perform the data collection. Among them, it has to be ensured that there is at least one meter between the device and the participants, and anything that can cover the participant face (her own hands, fringe, etc.) has to be removed.
- Tall participants had to look down to watch the monitor; this complicated the facial data collection. To solve this, the level of the monitor should be regulated, never forgetting that Kinect and webcam have to capture the whole face of the participant.
- When participants are people with low vision, usually they tend to get close to the computer screen. This makes the video capture more difficult as the face gets hidden behind the screen and cannot be completely recorded.
- Sometimes the expert informed about a lack of consistency between (1) the facial expression and body movements showed by the participant along the cognitive tasks, and (2) the information reported by them in the open answer emotional reports.
- Even though video-based techniques for emotion detection are considered as unobtrusively methods, some participants reported that they feel discomfort when a device is recording them.

In our view, the annotations done by experts after analyzing recorded videos in order to detect facial expressions and body movements and determine relations with the performed cognitive tasks will enrich other results obtained by the multimodal framework proposed in MAMIPEC project, such as those reported elsewhere [63]. In particular, data mining techniques are to be used to process and combine this affective labeling from face expressions and body movements with breath and heart rate measures, body temperature levels, galvanic skin response, keyboard and mouse interaction, and the data from the participants' emotional self-reports. The purpose here is to take into account the needs of all users, including those with disabilities [50]. Taking all this into account, the main objective of our research within the MAMIPEC project is to facilitate an affective support that allows learners to perform cognitive tasks by improving their emotional control strategies. In particular, we have discussed elsewhere [64] how to capture relevant information from an intelligent tutoring system that [65] focuses on one of the most challenging steps in learning algebra, which is the translation of word problems into symbolic notation, in order to improve the learner's competence in solving algebraic word problems considering learners' emotional and mental states.

**Figure 6:**
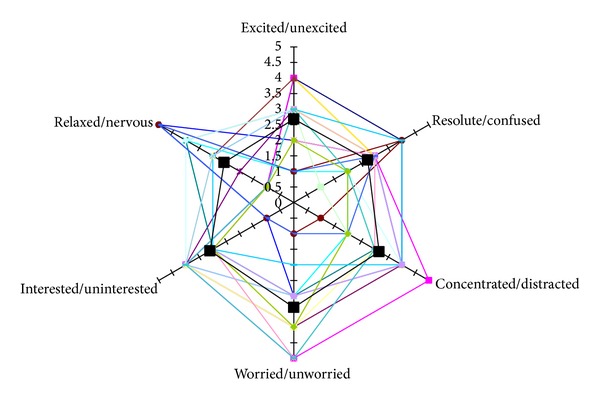
Values of the participants of the learning process categories.

The data analysis on the participants' behavior can be carried out in two orthogonal ways. One of them should focus on identifying relevant patterns within the set of information collected for all the participants as a whole. The other one should carry out a detailed analysis per participant. In order to select the most relevant participants for the analysis, we can select those who feature extreme values in the learning process categories considered in Section 4.1.3 regarding the participants' global interaction. In particular, using a radial representation Figure 6 shows the values for the different participants analyzed here (colored lines) which form diverse shapes in the figure, and the average value for all the participants (black line) which almost coincides with the middle values of the figure.

Additionally, based on the results of our experiments, we are also focused on following an intrasubject approach, where an exhaustive data collecting from a single user is to be done in order to model as precisely as possible this particular user's affective states along a large period in time.

## 6. Conclusions and Future Work

The purpose of this work was not to get conclusive results but to bear out the main challenges and difficulties involved in emotion detection from facial expressions and body movements in learning settings, aimed to enrich and support a multimodal framework for emotions detection in educational scenarios. In this sense, this paper is aimed at describing related background and the proposed approach for the annotation process, which includes the reported methodology to detect facial expressions, body movements, and associated emotional information when the learner is interacting in a learning environment involving cognitive tasks.

The annotation methodology that has been proposed considers data gathered from user's facial expressions and body movements (i.e., location, type, direction, intensity, and repetition); data gathered from the learning tasks (i.e., task, result, duration, and associated emotional reporting); and data gathered from participants' global interaction in terms of 6 categories (i.e., excited-unexcited, resoluted-confused, concentrated-distracted, worried-unworried, interested-uninterested, and relaxed-nervous).

The analysis showed that participants' emotional reactions in a real learning scenario seem to be influenced by the duration of the task, its difficulty level, and the valence and arousal levels reported, and this has an impact on the facial expressions and body movements done when carrying out learning tasks that involve cognitive process. However, in our analysis we still need to process and further analyze all the different sources of emotional data collected, which is part of our ongoing work. Nevertheless, in our view our current work has produced some contributions to the state of the art as we have proposed a methodology to support the annotation process of facial expressions and body movements that appear when learners carry out learning activities. This annotation methodology considers information about the cognitive process (which is very relevant in an educational scenario) associated with facial expressions and body movements. As far as we are aware, this is not considered in the literature of emotions detection, as discussed in Section 2. In our view, this proposal provides evidence on how human emotion recognition involves not only facial and body motion but should also consider other variables such as the cognitive process (memory, attention, etc.) developed by the subject, the contextual information provided by the environment, and so forth.

The emotional annotations and further coding obtained by applying the proposed methodology combined with the other emotional input data aforementioned in Section 3 is being used to identify affective recommendations needs in order to deliver personalized affective support when participants interact within e-learning platforms. Support is being designed according with the learner's emotion management needs and accessibility preferences when she is performing cognitive tasks with the help of the TORMES recommendations elicitation approach based on the ISO standard 9241-210 [66], extended to consider affective information [67]. A preliminary experiment to evaluate the impact of the adaptive and personalized affective support delivered to the learner during the learning experience has been carried out in 2013 Madrid Science Week [63]. The application of TORMES methodology has served to produce a scenario demanding emotional support, covered with 10 affective recommendations. These recommendations were positively validated by educational experts. In parallel, the data from the keyboard and mouse interactions is also being processed to detect behavior variations during the experiment, showing correlations between the average time between two mouse button press events or the standard deviation between two keystrokes and the valence dimension [59]. A further research, correlating the results from that study with the indicators extracted from this work needs to be done.

Furthermore, as previously anticipated, a tool that can help researchers during the manual annotation of the videos has been proposed by members of the aDeNu research group and is being developed in conjunction with the GTH research group (Speech Technologies Group) of the Polytechnic University of Madrid (Spain). Our requirements for this tool are the following. First, it should take as input the facial features and body movements extracted from the Kinect device using Microsoft Visual Studio API. Using the methods provided by this API, raw data should be codified through CANDIDE-3, and thereafter processed with the FACS method. The FACS classification obtained should be shown on the graphical user interface synchronized with the video and desktop recording, together with other external sources such as the learner physiological signals detected at the same time. Available algorithms for facial expression recognition process such as those enumerated in the related works section can also be integrated in this tool if relevant information is obtained. The tool should also allow the expert to annotate any affective information (with the methodology proposed here) in those moments of time that are needed to enrich the affective information detected.

A future research work will be to analyze the data collected in the collaborative learning scenarios mentioned in Section 3, which follow the Collaborative Logical Framework approach [48]. As aforementioned and detailed in [49], variables related to emotion expression in social context will be analyzed (i.e., interdependence of other people, level of social competence, anxiety related with being better than others, etc.). Collaborative scenarios are of relevance as they provide information about cognitive process and strategies specifically involved in social contexts such as communication, clarification, and handling criticism abilities. Moreover, in future works we will also analyze the particularities for the emotions annotation when learners have some kind of disability.

## Figures and Tables

**Table 1:** Movements detected per task and task duration, as well as average per participant.

	T3	T4	T5
Movements	385	180	184
Duration (seconds)	9944	3697	6505
Ratio movements/duration	0.0387	0.0487	0.0283
Avg. movements/participant	20.26	9.47	9.68
Avg. duration/participant	523.37	194.58	342.37
